# Plasma Ghrelin Concentrations Were Altered with Oestrous Cycle Stage and Increasing Age in Reproductively Competent Wistar Females

**DOI:** 10.1371/journal.pone.0166229

**Published:** 2016-11-09

**Authors:** Michelle L. Johnson, M. Jill Saffrey, Victoria J. Taylor

**Affiliations:** School of Life, Health and Chemical Sciences, Faculty of Science, Technology, Engineering and Mathematics, The Open University, Milton Keynes, United Kingdom; Max Delbruck Centrum fur Molekulare Medizin Berlin Buch, GERMANY

## Abstract

Changes in appetite occur during the ovarian cycle in female mammals. Research on appetite-regulatory gastrointestinal peptides in females is limited, because reproductive changes in steroid hormones present additional experimental factors to control for. This study aimed to explore possible changes in the orexigenic (appetite-stimulating) gastrointestinal peptide hormone ghrelin during the rodent oestrous cycle. Fed and fasted plasma and stomach tissue samples were taken from female Wistar rats (32–44 weeks of age) at each stage of the oestrous cycle for total ghrelin quantification using radioimmunoassay. Sampling occurred during the dark phase when most eating takes place in rats. Statistical analysis was by paired-samples *t*-test, one-way ANOVA on normally distributed data, with Tukey post-hoc tests, or Kruskal-Wallis if not. GLM univariate analysis was used to assess main effects and interactions in ghrelin concentrations in the fed or fasted state and during different stages of the ovarian cycle, with age as a covariate. No consistent fed to fasted ghrelin increases were measured in matched plasma samples from the same animals, contrary to expectations. Total ghrelin concentrations did not significantly change between cycle stages with ANOVA, in either fed or fasted plasma or in stomach tissue. This was despite significantly decreased fasted stomach contents at oestrus (*P* = 0.028), suggesting decreased food intake. There was however a significant interaction in ghrelin plasma concentrations between fed and fasted proestrus rats and a direct effect of age with rats over 37 weeks old having lower circulating concentrations of ghrelin in both fed and fasted states. The biological implications of altered ghrelin plasma concentrations from 37 weeks of age are as yet unknown, but warrant further investigation. Exploring peripheral ghrelin regulatory factor changes with increasing age in reproductively competent females may bring to light potential effects on offspring development and nutritional metabolic programming.

## Introduction

Ghrelin is a key gut peptide involved in eating behaviour that is secreted predominantly from X/A-like cells of the stomach [1]. Ghrelin exists in two forms, an acylated and de-acylated (des-acyl) form. Ghrelin effects are mediated by the acylated form of the peptide and its receptor GHSR1a, to which the des-acyl ghrelin form does not bind. The GHSR1a receptor is found in the pituitary, other brain regions and body organs, including the pancreas and ovary [2]. To date, ghrelin is the only orexigenic (appetite-increasing) peptide to have been isolated that is produced outside the central nervous system, and its role in whole body energy homeostasis and feeding stimulation is a major focus of research, especially for its potential control of body mass within healthy ranges. New drugs and endogenous hormone combinations are being tested as alternatives to risky gastric surgery treatments that are effective in altering gut hormone profiles towards weight loss [3]. The increasing incidence of obese reproductive age females in human populations across the globe [4] is of concern for the health of these women and their offspring, so it is essential that we understand more about the role of gut peptides in normal eating behaviour and reproductive functioning. Ghrelin concentrations are highest in the circulation prior to feeding episodes, after which they reduce and then plateau [5].

Research linking food intake and reproductive cycle stage has been published on rodents, pigs, goats, sheep, primates and humans [6]. The established link between oestrogens and food intake has been shown to result in a reduced food intake leading up to ovulation, due to the latent effects of an earlier ovarian peak in oestradiol secretion [7]. In rodents, reduced food intake occurs during proestrus [7], but it is unknown whether changes in peripheral ghrelin concentrations also occur at this time. This is despite it being established that ghrelin plays a facilitative role in successful fertilisation, implantation and early embryo development in mice [8].

Although previous studies have established that there are potentially reciprocal relationships between oestradiol [9, 10], the reproductive cycle and ghrelin, no studies have yet quantified ghrelin concentration in both fed and fasted plasma and in gut tissue from rats allowed to cycle normally. Rats are considered to be a good model for this type of study, as their size allows sampling of plasma and organ tissues in adequate amounts for in-depth analysis of multiple tissues, matched samples and simultaneously circulating hormone concentrations. It may be anticipated that during oestrus, when food intake is lowest, ghrelin levels may be attenuated due to the peak of oestradiol concentrations during proestrus and may contribute to the anorexigenic tone.

Due to the limited previous research conducted on appetite-regulatory gastrointestinal peptides in females, because steroid hormones present additional experimental factors to control for, the present study aimed to investigate whether circulating and stomach tissue total ghrelin concentrations change in line with reported appetite changes during the oestrous cycle, in rats aged 38 ± 0.49 weeks (32–44 weeks). It was hypothesised that a reduction in food intake may be seen prior to oestrus, with a concurrent decrease in total ghrelin levels, however this simple relationship was not found. There was a significant interaction in ghrelin plasma concentrations between fed and fasted proestrus rats and additionally, rats over 37 weeks old were found to have lower circulating concentrations of ghrelin in both fed and fasted states. This is the first study to consider ghrelin concentrations in the dark (eating) phase in matched fed and fasted plasma and in corresponding stomach tissue samples in female rats at each stage of the oestrous cycle.

## Materials and Methods

### Animals

This work was licensed under the Home Office Animals (Scientific Procedures) Act 1986 and had approval from The Open University Ethics Committee. Rats were chosen to obtain sufficient blood for matched fed and fasted circulating hormone concentrations and stomach tissue peptide comparisons. Nulliparous female Wistar rats (Harlan, UK; n = 43) were housed in groups of four and maintained under a 12 hour reverse light cycle (lights off between 11.00 and 23.00), with free access to standard diet, water and bedding material. All procedures were carried out during the dark phase so that samples were obtained when most physiologically relevant for natural feeding behaviour. Females were kept near a cage of male rats to keep the females cycling normally. Daily oestrous monitoring was undertaken at 24-hourly intervals between 11.00 and 13.00 to determine cycle stages by vaginal lavage [11]. Rats were between 32–44 weeks of age and had a body mass range of 239.6–303.9 g. There were four experimental groups in total, one for each stage of the oestrous cycle, and females were chosen at random to fulfil group numbers. Some animals did not progress as predicted with their oestrous cycle, so there is a mismatch between the number of animals in the fasted state cycle stages (proestrus n = 12; oestrus n = 11; metoestrus n = 9, dioestrus n = 11) and the numbers obtained for the fed cycle stages the day before (proestrus n = 14; oestrus n = 7; metoestrus n = 6, dioestrus n = 16).

### Blood Collection and Preparation

Fed blood samples from a tail vein were taken between 13.00 and 15.00, after completion of oestrous monitoring, and animals were returned to their home cage in their established social groups. Topical analgesia (Cryogesic, Ethyl Chloride BP fine spray, Acorus Therapeutics Ltd) was applied to minimise pain at the withdrawal site. Cage food was removed (free access to water was maintained) at 08.00 the following day prior to sacrifice between 12.00 and 16.00. Rats were anaesthetised (isofluorane; IsoFlo, Abbott) and decapitated, and a fasted blood sample was obtained from trunk blood. All blood was collected into EDTA coated tubes with additional protease inhibitor (aprotinin; Trasylol, Bayer). Blood samples were immediately acidified by dilution at 1:10 in buffer (0.1 M ammonium acetate, 0.5 M NaCl, pH 3.6) as recommended for optimal ghrelin peptide preservation and recovery [12].

### Stomach Tissue Collection and Preparation

Stomachs were removed and masses were recorded both before and after opening along the greater curvature and rinsing in PBS. Mass of remaining stomach contents was also calculated. Half of each rat stomach was collected and immediately frozen. Stomach samples were extracted in 1 ml of 0.5 M glacial acetic acid per 100 g of tissue collected, and boiled in a 100°C water bath for 20 minutes. The liquid portion of the boiled samples was stored at -20°C until assayed.

### Radioimmunoassay

Samples of fed and fasted acidified plasma and stomach tissue extract were analysed in duplicate and according to the manufacturer’s protocol for total ghrelin concentration using radioimmunoassay kits (Millipore, UK). Preliminary testing measured high concentrations of ghrelin in all samples, so they were diluted ten times by a reduction in sample volume in the assay tubes. Stomach tissue extracts underwent an additional 1:400 dilution with distilled water. The intra-assay coefficient of variation was 10.7% and the inter-assay coefficient of variation was 4.7%, across three assays of the same assay batch number. Samples were added to assays based on sample type, not cycle stage.

### Statistical Analysis

Values represent mean ± S.E.M. Initial statistical analysis was carried out using a one-way ANOVA with a Tukey post-hoc test on normally distributed data. When data were not normally distributed and could not be normalised (e.g. log transformation), a Kruskal-Wallis test was used. A paired-samples *t*-test was used to compare ghrelin in the fed and the fasted state, and a Mann-Whitney U test was used to analyse stomach contents across age groups, as these data were not normally distributed. Further statistical testing used a general linear model (GLM) univariate analysis with ghrelin concentration as the primary dependent (within subject) variable and fed/fasted status and stage in cycle as independent (between subject) variables, with age in weeks as a covariate, to test for main (direct) and interaction effects. To evaluate our secondary aims, Pearson correlations were used to determine if ghrelin concentrations were correlated in fed and fasted states and within different age groups. All statistical tests were performed using IBM SPSS Statistics 21. *P*<0.05 was considered statistically significant.

## Results

There were no significant differences in age (*P* = 0.180) or body mass (*P* = 0.673) between the different cycle stage groups using ANOVA.

### Fasted Stomach Contents Indicated Least Food Consumed Leading Up to Oestrus

**Fig 1:**
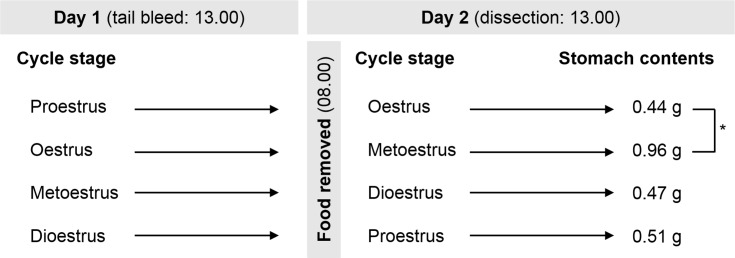
The mass of fasted stomach contents after each stage of the oestrous cycle. The reduced stomach contents seen after proestrus/oestrus was significantly less than after oestrus/metoestrus, indicating reduced earlier food and water consumption. (Cull cycle stages: proestrus, n = 12; oestrus, n = 11; metoestrus, n = 9; dioestrus, n = 11. *P<0.05).

Stomach contents data were analysed against the cycle stage of the preceding day, as the rats were fasted from the beginning of the cull day (from 08.00; before lights off at 11.00), therefore analysis of remaining stomach contents provided an indication of food consumption during the previous day/cycle stage (Fig 1). Rats dissected at metoestrus, so fasted from oestrus, had significantly (*F*(3, 39) = 3.187, *P* = 0.034) more (fasted) stomach contents (0.96 g) than those dissected at oestrus (fasted from proestrus; 0.44 g; *P* = 0.028). There was a negative correlation between fasted remaining stomach contents and stomach tissue ghrelin concentrations (*r* = -0.305, *P* = 0.047, n = 43).

### Ghrelin Concentrations during the Oestrous Cycle

Separate (fed or fasted only) data analysis did not reveal any significant differences between the concentrations of ghrelin in fed acidified plasma (Kruskal-Wallis, χ2 = 4.153, 3 df, *P* = 0.245; Table 1) or in fasted acidified plasma ghrelin (Kruskal-Wallis, χ2 = 7.556, 3 df, *P* = 0.056; Table 1) between cycle stages. There were also no significant differences found in concentrations of ghrelin in stomach tissue extracts between each cycle stage (*F*(3, 39) = 0.782, *P* = 0.511; Table 1) by parametric analysis.

**Table 1:** Concentrations of ghrelin in fed and fasted plasma and in fasted stomach tissue during the oestrous cycle.

Cycle Stage	Fed plasma	Fasted plasma	Stomach tissue
	(ng/ml)	(ng/ml)	(μg/g WWT*)
**Proestrus**			
Mean	202.3^a^	148.6^b^	12.5
S.E.M	12.92	4.54	0.65
*n*	14	12	12
**Oestrus**			
Mean	173.1	188.6	14.3
S.E.M	12.03	13.88	1.20
*n*	7	11	11
**Metoestrus**			
Mean	187.4	184.4	12.4
S.E.M	3.66	13.18	1.09
*n*	6	9	9
**Dioestrus**			
Mean	160.6	179.0	14.4
S.E.M	6.85	11.17	1.84
*n*	15	11	11

GLM univariate, a>b, *P* = 0.014.

* μg per g of wet weight of tissue.

**Fig 2:**
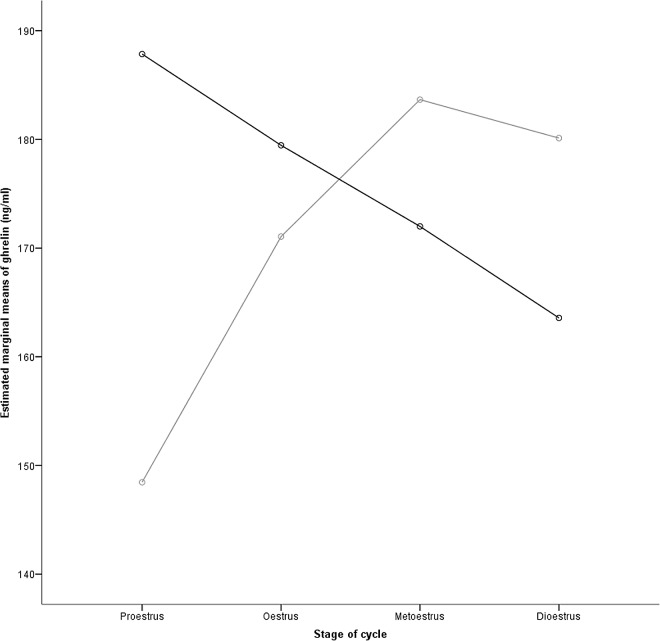
Interaction between fed and fasted plasma concentration of total ghrelin during the oestrous cycle. Proestrus fed ghrelin (black line) concentrations were significantly (*P* = 0.014) higher than proestrus fasted (grey line) concentrations.

Significant total ghrelin plasma concentration differences were found in relation to ovarian cycle stage and fed/fasted status in a GLM univariate analysis. There was a significant main (direct) effect of age factor (*F*(1,69) = 65.211, *P*<0.001) and a significant interaction (joint effect) of fed/fasted status with ovarian cycle stage fixed factors (*F*(6,69) = 4.149, *P* = 0.001), and also when age was included in the model as a covariate (*F*(7,69) = 3.353, *P* = 0.004). Although ghrelin did not differ consistently between fed and fasted conditions, there was a different pattern (Fig 2) demonstrating an interaction between fed/fasted status and ovarian cycle stage, with proestrus fed concentrations higher than proestrus fasted concentrations (Table 1, *P* = 0.014), consistent with decreased feed intake prior to oestrus.

### Plasma Ghrelin Concentrations Were Not Consistently Different following Fasting

There was no significant difference (*t*(41) = 2.015, *P* = 0.051; n = 42) between the concentrations of peripherally circulating ghrelin in fed (180.4 ± 6.66 ng/ml) and fasted (174.3 ± 5.97 ng/ml) states, which was unexpected.

As each rat was progressing from one to the next ovarian cycle stage between the paired fed and fasted blood samples, to determine if the expected fasting ghrelin concentration increase took place, ratios of the mean values from Table 1 were calculated: proestrus fed/oestrus fasted = 1.08, oestrus fed/metoestrus fasted = 0.94, metoestrus fed/dioestrus fasted = 1.05, dioestrus fed/proestrus fasted = 1.08. Only rats transitioning from oestrus to metoestrus had higher mean fasted ghrelin concentrations. They also had most remaining stomach contents (Fig 1) suggesting that they ate more.

**Fig 3:**
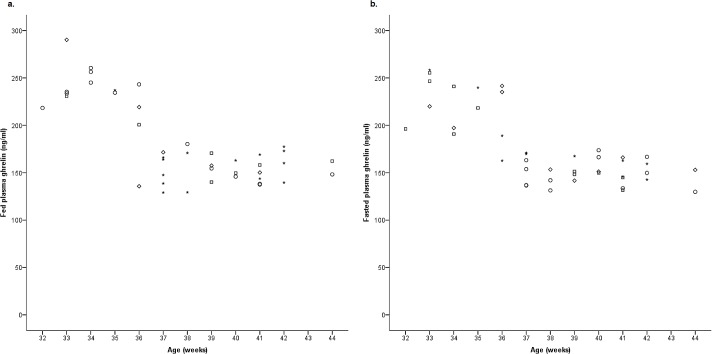
Correlation of plasma ghrelin concentrations with rat age. Plasma ghrelin concentrations negatively correlated with age (fed, *r* = -0.715, *P*<0.001, n = 42) (fasted, *r* = -0.744, *P*<0.001, n = 43). (○ proestrus; □ oestrus; ◊ metoestrus; * dioestrus).

**Fig 4:**
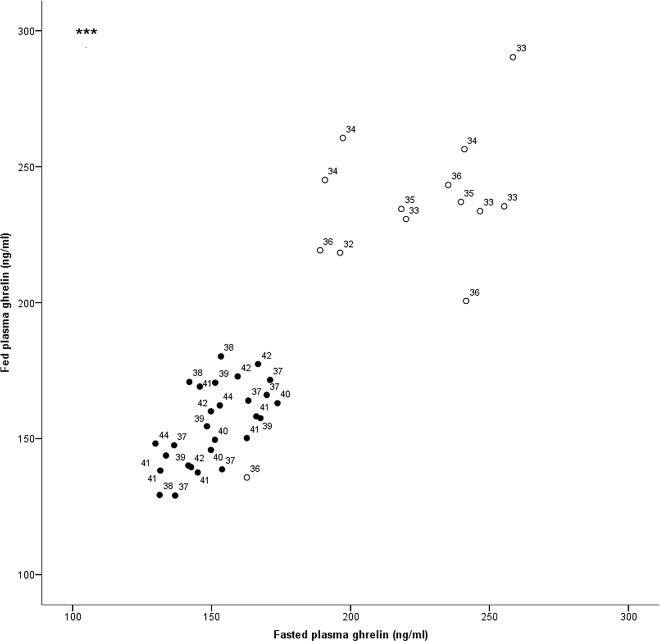
Correlation of plasma ghrelin concentrations in the fed and fasted states with rat age. Fed and fasted ghrelin concentrations correlated positively with each other (*r* = 0.890, *P*<0.001, n = 42). (○ 32–36 weeks, n = 14; ● 37–44 weeks, n = 28; *** *P*<0.001; one-way ANOVA, Tukey post-hoc). Numbers on figure against each data point represent age in weeks, younger animals (<37 weeks) had higher ghrelin concentrations.

Fed and fasted plasma ghrelin concentrations negatively correlated with age (fed, *r* = -0.715, *P*<0.001, n = 42, Fig 3A; fasted *r* = -0.744, *P*<0.001, n = 43, Fig 3B), and positively correlated with each other (*r* = 0.890, *P*<0.001, n = 42; Fig 4).

### Plasma, but Not Stomach Tissue Ghrelin Concentrations Were Different with Age

General linear modelling analysis revealed a significant direct effect of age, and scatterplots of the data showed that plasma ghrelin concentrations were lower in older animals, with those from 37–44 weeks of age having significantly (fed: *F*(1, 40) = 101.77, *P*<0.001; fasted: Kruskal-Wallis, χ2 = 25.416, 1 df, *P*<0.001) reduced circulating ghrelin (fed: 154.8 ± 2.77 ng/ml; fasted: 151.5 ± 2.45 ng/ml) than those from 32–36 weeks of age in both the fed (231.5 ± 9.30 ng/ml) and the fasted (220.9 ± 7.82 ng/ml) states (Fig 4). There was one exception, see discussion. The slightly older animals with less circulating ghrelin had a stronger positive correlation between fed and fasted concentrations *(r* = 0.619, *P*<0.001, n = 28) than the younger animals (*r* = 0.583, *P* = 0.029, n = 14). Moreover, nearly half (48.3%), of the older rat group had a higher fasted plasma ghrelin concentration than fed, as expected, however only 35.7% of the younger animals displayed this relationship.

Stomach tissue ghrelin concentration was not significantly different between the different age groups (32–36 weeks: 15.2 ± 1.58 μg/g WWT; 37–44 weeks: 12.6 ± 0.49 μg/g WWT; *P* = 0.076, ns). There was no difference in body mass between the older and younger groups (*F*(1, 41) = 0.070, *P* = 0.793). In addition, there was no difference in the mass of fasted stomach contents the age groups (32–36 weeks: 0.6 ± 0.15 g; 37–44 weeks: 0.5 ± 0.06 g; *P* = 0.736, ns).

## Discussion

This study aimed to elucidate the role of the gut hormone ghrelin in reported changes to appetite during the oestrous cycle in rats and is the first study to explore the concentrations of matched fed and fasted plasma and corresponding stomach tissue ghrelin concentrations at each stage of the oestrous cycle with samples taken during the dark (eating) phase.

The concentrations of total ghrelin did not change significantly with the stage of the cycle in either fed or fasted plasma or in stomach tissue extracts when analysed separately by ANOVA or Kruskal-Wallis, despite observed differences in fasted stomach contents following oestrus. On the surface, these combined data could suggest that total ghrelin concentration fluctuations are not implicated in the appetite changes known to occur prior to oestrus in rats, and/or that circulating total amounts of ghrelin are tightly regulated. However, GLM univariate analysis, revealed significant joint effects of fed/fasted status and cycle stage on plasma total ghrelin concentrations in proestrus rats which suggests altered regulation of ghrelin between fed and fasted states close to oestrus (and ovulation).

The remaining mass of fasted stomach contents in these animals provides evidence of an adequate fasting time, and suggests that animals consumed the least between proestrus and oestrus and the most shortly after the end of oestrus leading into metoestrus, as has been documented by others [7]. The ratios of fed to fasted ghrelin as rats transitioned between ovarian cycle stages were similar except for from oestrus to metoestrus, the only time when mean fed ghrelin concentrations were lower than fasted, consistent with the suggestion that the rats were hungrier/ate more at this time. Differences in fasted stomach contents found between cycle stages could alternatively indicate a different rate of gastric emptying, which was not measured.

The experimental fasting regime was adequate to reveal any differences due to feeding and fasting as concentrations of anorexigenic gastrointestinal appetite hormones peptide-YY and glucagon-like peptide-1 measured from the same plasma samples did show expected significantly different fed and fasted responses (unpublished data: Johnson *et al*. in prep.). The animals in the present study were on a reverse lighting schedule and had plasma acidified for ghrelin analysis. Both of these factors could have revealed different ghrelin concentrations in comparison to previous studies, which have not taken these steps to optimise both the physiological timing (rodents mainly feed in the dark) of sample collection or optimal ghrelin peptide recovery. In addition to the findings from this study, the possibility remains that the proportions of the different forms of ghrelin [13] (acyl, desacyl) could also be altered and requires further study to determine

An unexpected finding from this study was that plasma ghrelin concentrations between the fed and the fasted state were not found to be significantly different, which is contrary to previous reports, but our results suggest that, at least in females, ovarian cycle stage may have a further influence. Furthermore, it was surprising, especially when similar age and body mass rats were used, that plasma ghrelin levels were reduced in both the fed and fasted state by 37 weeks of age. Only one 36 week old animal was an exception, with a low concentration of circulating ghrelin. This particular female had the highest amount of ghrelin peptide in her stomach tissue (31.9 μg/g WWT) compared with the other animals (group mean 13.4 ± 0.63 μg/g WWT), so sampling may have occurred during production and storage of gastric ghrelin rather than after release into circulation.

The slightly older animals had a much tighter correlation between their fed and fasted plasma ghrelin concentrations than the younger animals and more of them displayed increased total ghrelin concentrations prior to anticipated feeding time (as would be expected based on other earlier studies). Fewer of the younger animals had the expected relationship between fed and fasted ghrelin, with only 35.7%, compared with nearly half of the older rats, having a higher fasted ghrelin concentration. These findings remain to be explained, but could indicate a difference in the way that ghrelin regulated appetite and/or whole body energy homeostasis between the younger and the older female animals, with ghrelin release differentially regulated by other additional factors prior to 36 weeks of age.

Differences in secretion of reproductive hormones due to increasing age may relate to the disparity found between ghrelin concentrations in the older and younger animals. However, this is unlikely, for two main reasons. First, oestradiol has an inhibitory effect on endogenous ghrelin concentrations in circulation [7], so declining levels of circulating oestradiol with increasing age are unlikely to be responsible for the unexplained observed decrease in plasma ghrelin concentrations. Second, reproductive ageing occurs many months later than the age of the rats used in this study, so it is unlikely that declining reproductive function leading to reducing oestradiol concentrations was a major factor in the notable reduction of plasma ghrelin levels in the present study. The reduced circulating total ghrelin differences measured in matched fed and fasted plasma samples occurred from approximately eight to nine months of age, with no parallel changes in stomach ghrelin peptide concentrations. Reproductive senescence is not expected to occur until between approximately 15 and 20 months of age in laboratory rats [14], which is when measurable changes (increases) in ghrelin levels could be anticipated.

It is more likely that other and/or local factors may be involved in the observed ghrelin changes. In rats, oestrogen secreted from the gastric mucosa has been shown to induce stomach ghrelin expression [15], although a further study found that gastric oestrogen was not responsible for the fasted increase of ghrelin mRNA expression [16]. Future studies could undertake further investigation into oestrogen concentrations in stomach tissue as these may provide insights into whether reproductive ageing may cause a decrease in gastric oestrogen, which could in turn cause a blunted stomach ghrelin production in response to food intake, potentially lowering circulating concentrations.

One study that provides the most comprehensive picture of the inhibitory effect of oestradiol on ghrelin in rats demonstrated that infusion of ghrelin increased food intake in males and in OVX females, but not in either oestrogen-treated males or intact females, suggesting that oestrogen somehow attenuates ghrelin’s ability to stimulate appetite [9]. In OVX females treated with cyclic oestradiol to mimic the oestrous cycle, ghrelin infusion increased food intake on the equivalent days of metestrus and diestrus (low oestrogen levels), but had no effect during proestrus and oestrus (high oestrogen levels), which was also replicated in normally cycling rats. Butera’s review of the work in their lab also concluded that injection of ghrelin during diestrus, but not proestrus, increased food intake and that this occurred due to increased meal frequency but not individual meal size [17]. These findings suggest that during times of high oestradiol (proestrus and oestrus), endogenous ghrelin levels may be reduced, or ghrelin’s actions could be attenuated by high oestradiol levels. The finding of increased meal frequency but not meal size during diestrus is contrary to what Eckel *et al*. [18] reported where there was a decrease in meal size but not frequency at oestrus. Taken together, these studies may suggest that independent mechanisms are responsible for the increase in food intake at diestrus and the decrease in food intake at oestrus. Future studies would aim to measure both circulating oestradiol and stomach oestrogen, and to examine the ovaries of naturally cycling females to further elucidate the mechanisms of the findings obtained in this study.

Understanding the regulation of ghrelin levels at different stages of reproductive life is important, because of the potential effect on the developing offspring, from developmental stages through to adulthood. For example, a study using ghrelin heterozygous mice reported that a systemic low level of maternal ghrelin appeared to have an adverse effect on the uterine programming of their wild-type female offspring, causing subnormal fertility rates through reduced implantation, despite normal ovarian function and embryo production [19]. These authors suggested that their findings could have serious implications for the fertility of women born to obese mothers who would have had blunted ghrelin levels due to their obesity. Another recent study has highlighted that a maternal high fat diet during pregnancy and lactation influenced both ghrelin and obestatin levels in the dams themselves, and their offspring [20]. These findings provide further support for the need to study females throughout their ovarian cycles, as well as during pregnancy, lactation and beyond, as pre-conception events are also critical to healthy reproductive outcomes.

This study provides evidence that circulating total ghrelin concentrations are influenced by complex physiological interactions taking place between fed and fasted states and stages of the ovarian cycle in female rats, that are also influenced by age (within a relatively narrow range and whilst still reproductively competent). The present results indicate that other factors, including circulating oestradiol and stomach oestrogen levels, require further investigation for possible effects on circulating total ghrelin concentrations in young adult females.

## Conclusions

Significant changes were detected in total ghrelin concentrations in fed and fasted plasma, but not in stomach tissue, during different stages of the oestrous cycle in Wistar rats, along with observed decreased fasted stomach contents at oestrus. Fasted plasma ghrelin concentration increases were only observed in matched fed samples when rats were transitioning from oestrus to metoestrus on consecutive days. Circulating ghrelin levels were significantly reduced by 37 weeks of age in rats in both fed and fasted conditions. The biological implications of these findings are yet to be established in reproductively active animals, but warrant further investigation into ghrelin regulatory factor changes with increasing age in reproductively competent female mammals. If replicated in humans, the response to feeding, and higher circulating concentrations of total ghrelin in younger adult females, have the potential to influence the findings and interpretation of other studies, potential drug treatments, and surgical interventions. Future studies would need to factor ovarian cycle stage and age-related ghrelin regulatory changes into their experimental design and data analyses. Likewise, with the shift towards older human mothers with potentially altered concentrations of appetite-influencing hormones, producing their first children later in life, may also affect development in utero and post-partum nutrition, thus the metabolic programming of future generations.
